# Breath acetone as a marker of energy balance: an exploratory study in healthy humans

**DOI:** 10.1038/s41387-018-0058-5

**Published:** 2018-09-10

**Authors:** Fabian Bovey, Jérémy Cros, Béla Tuzson, Kevin Seyssel, Philippe Schneiter, Lukas Emmenegger, Luc Tappy

**Affiliations:** 1 Department of Physiology, Faculty of Biology and Medicine, University of Lausanne, Lausanne, Switzerland; 2 Empa, Laboratory for Air Pollution/Environmental Technology, Überlandstrasse 129, 8600, Dübendorf, Switzerland; 3 Cardio-Metabolic Center, Broye Hospital, Estavayer-le-lac, Switzerland

## Abstract

An exploratory study was performed on eight healthy volunteers to assess how short-term changes in energy balance and dietary carbohydrate content impact breath acetone concentrations. Participants were studied on three occasions: on each occasion, they remained fasted and in resting conditions during the first 2 h to assess basal breath acetone and blood beta-hydroxybutyrate (BOHB). During the next 6 h, they remained fasted on one occasion (F), or were fed hourly high carbohydrate (HC) or low-carbohydrate (LC) meals to induce a positive energy balance on the other two occasions. They remained in resting conditions during 4 h, then performed a 2-hour low intensity exercise (25 W) inducing a negative energy balance. In resting conditions, breath acetone and blood BOHB concentrations increased progressively compared to basal values in F, but decreased and remained low throughout the test in HC. With LC, breath acetone increased progressively, while blood BOHB decreased. This exploratory study indicates that breath acetone reliably detects a stimulation of ketogenesis during a short-term fast. It also suggests that LC and HC differentially impact BOHB and acetone production and utilization, and reveals possible limitations to the use of breath acetone as a marker of energy balance.

## Introduction

The treatment of obesity consists in inducing a negative energy balance over several months or years. The rate of success with lifestyle interventions is disappointingly low, however, possibly because many patients fail to achieve a negative energy balance by overestimating their physical activity and underestimating their energy intake^1^. In this regard we^2^ and others^3,4^ have proposed that monitoring breath acetone as a marker of energy balance may be a useful tool to improve lifestyle intervention efficiency. Ketogenesis is indeed stimulated by fasting^5^, caloric restriction^6^, and exercise^7^. However, it can also be stimulated during consumption of a very low-carbohydrate diet independently of negative energy balance^8,9^. Whether increased ketogenesis can be detected from breath acetone measurements when energy balance is moderately negative, and whether this is influenced by dietary carbohydrate content, remains however unknown. We therefore monitored breath acetone, using a recently developed laser based breath analyzer^10^, in healthy volunteers after an overnight fast followed by a 4-hour period during which volunteers remained fasted or were fed hourly meals with either 70% or 10% carbohydrate. Thereafter, a 2-h low intensity exercise was performed.

## Methods and procedures

Eight healthy volunteers (4 men and 4 women, mean age ± SEM: 26 ± 2 years; weight: 67.3 ± 4.0 kg; body mass index: 22.5 ± 0.6 kg·m^−2^) were recruited. All subjects were weight-stable, non-smokers, and had no personal or family history of diabetes. The protocol was approved by the Human Research Committee of Canton de Vaud and was registered at clinicals.gov (NCT03390881) and participants provided an informed, written consent.

Participants were studied on three different occasions in a randomized, open-label, cross-over design. The two days before each test, participants consumed their usual diet and performed minimal physical activity. At 08:00 PM the day before, they received a standardized meal covering 30% of their calculated energy requirements (55% carbohydrate, 30% lipid and 15% protein).

On the test day, subjects came to the laboratory at 07:00 AM in fasting state. Subjects remained fasting while lying in a bed for an initial two-hour period and three breath acetone and blood BHOB measurement were obtained to determine basal values. Thereafter, and for the next 6 h (T120 to T480), they either remained fasted (F), or received every hour a liquid meal providing 150% of their resting 1-h energy requirement (1.5 times their RMR times 60 min) with either 70% carbohydrate (30% sucrose (Hänseler Swiss Pharma) and 40 % maltodextrin (Sponser, Switzerland)), 15% lipid and 15% protein (high carbohydrate; HC), or 70% lipid, 15% protein and 15% sucrose (low carbohydrate; LC). They remained in resting conditions from T120 to T360, then biked at 25 W from T360 to T480. Breath and blood samples were obtained every hour, while respiratory gas exchange was monitored by indirect calorimetry (Cosmed Quark RMR, Cosmed, Roma, Italy).

Breath samples were collected in air bags (Cali-5-Bond^TM^ bags, Calibrated Instruments Inc., Garrett Highway, USA) after a 5-s apnea and after having discarded the respiratory dead space^3^. Breath acetone was measured within 12–24 h using a custom-developed laser spectrometer (VECSEL, Camlin Technologies, Zurich, Switzerland), as described^10^. Since basal values showed considerable inter-individual [between-subjects coefficient of variation (CV) = 36%] and intra-individual (between test CV in the same subject = 32%) variations, breath acetone concentration at T120 was used as a reference for each subject. Plasma non-esterified fatty acid (NEFA), BOHB and glucose were measured using enzymatic methods (Randox Laboratories, Crumlin, UK), and plasma insulin by radio-immunoassay (Millipore Corporation, Billerica, MA, USA).

All values were expressed as mean ± SEM. Sample size was arbitrarily set at 8. The normality and homoscedasticity of the distributions were checked by Shapiro-Wilk and Bartlett tests. When needed, variables were normalized using the Box-Cox transformation. Changes in variables were assessed with a one-factor analysis of variance (ANOVA). Multiple comparisons were then performed by Student’s paired *t* tests. Linear associations between changes in breath acetone and plasma BOHB were tested using Spearman’s correlation. Data were analyzed using “R”, version 3.3.1 (www.cran.R-project.org).

## Results

### Basal conditions

Mean breath acetone concentrations were 2.04 ± 0.21 ppm and blood BOHB concentrations were 0.13 ± 0.02 mmol L^−1^.

### Fasting condition

**Fig. 1:**
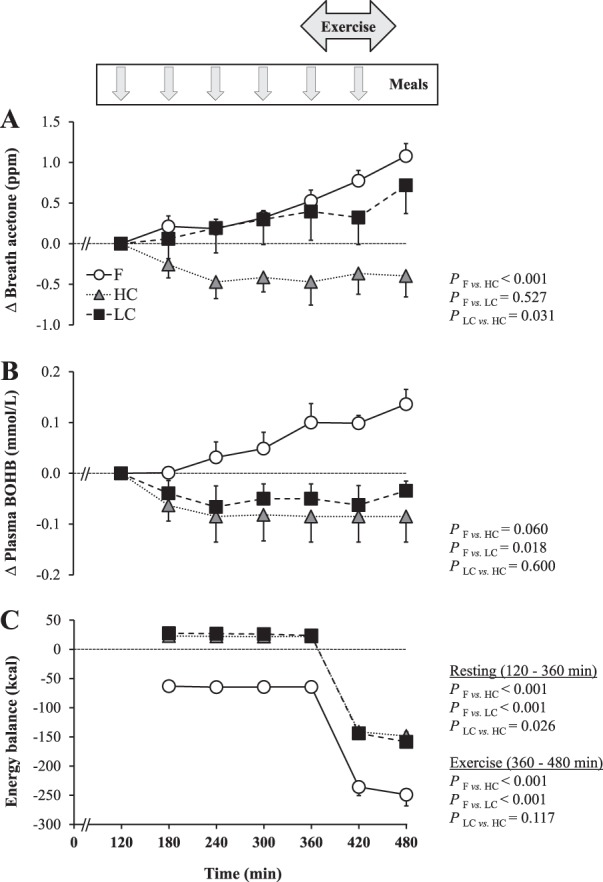
Changes in breath acetone (**a**) and plasma beta-hydroxybutyrate concentrations (**b**), and energy balance (**c**) when participants remained fasted (**f**) or were fed hourly with high- or low-carbohydrate meals. Data are expressed as mean ± SEM. For all variables, *n* = 8 volunteers. BOHB beta-hydroxybutyrate, F fasting, HC high carbohydrate, LC low carbohydrate

**Fig. 2:**
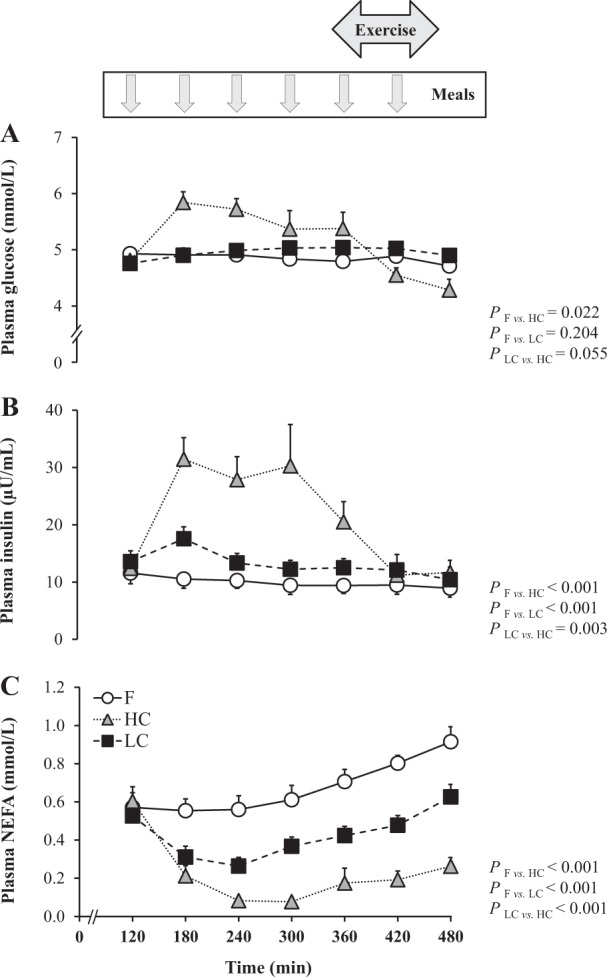
Changes in plasma glucose (**a**), insulin (**b**) and NEFA concentrations (**c**) when participants remained fasted (**f**) or were fed hourly with high- or low-carbohydrate meals. Data are expressed as mean ± SEM. For all variables, *n* = 8 volunteers. F fasting, HC high carbohydrate, LC low carbohydrate, NEFA non-esterified fatty acid

Breath acetone concentrations increased progressively compared to basal values, and reached their maximum at the end of the exercise. In parallel, plasma BOHB concentrations increased continuously over time, without any detectable acceleration during exercise. Changes in breath acetone were positively correlated with changes in BOHB (ρ_36_ = 0.58, *P* < 0.001). Fat oxidation rate averaged 1.13 ± 0.06 mg kg^−1^ min^−1^ at rest and increased to 4.39 ± 0.25 mg kg^−1^ min^−1^ during exercise. Energy expenditure corresponded to 1.07 ± 0.06 kcal min^1^ at rest and 4.04 ± 0.28 kcal min^−1^ during exercise. Energy balance was negative throughout the test (Fig. 1). Plasma glucose and insulin concentrations showed no changes over time, but plasma NEFA concentrations increased progressively (Fig. 2).

### High-carbohydrate meals vs. fasting

When subjects consumed HC meals, breath acetone and plasma BOHB concentrations decreased slightly, reaching lower levels than in F. No correlation was found between changes in breath acetone and BOHB (ρ_23_ = −0.15, *P* = 0.487). The fat oxidation rate was significantly lower than in F both at rest (0.94 ± 0.04 mg kg^−1^ min^−1^, *P* = 0.002) and during exercise (2.07 ± 0.30 mg kg^−1^ min^−1^; *P* < 0.001). Energy expenditure corresponded to 1.14 ± 0.07 kcal min^−1^ at rest and 3.93 ± 0.19 kcal min^−1^ during exercise. Energy balance was slightly positive at rest and negative during exercise (Fig. 1). Plasma glucose and insulin concentrations were significantly higher, while plasma NEFA were significantly lower than in F (Fig. 2).

### Low-carbohydrate meals vs. fasting

When subjects consumed LC meals, breath acetone increased progressively over time, and showed no significant difference compared to F. In contrast, plasma BOHB was significantly decreased. Changes in breath acetone were not correlated with changes in BOHB (ρ_16_ = −0.34, *P* = 0.164). Fat oxidation rate averaged 1.16 ± 0.06 mg kg^−1^ min^−1^ at rest (*P* = 0.727) and 3.79 ± 0.20 mg kg^−1^ min^−1^ during exercise (*P* = 0.013). Energy expenditure corresponded to 1.13 ± 0.07 kcal min^−1^ at rest and 4.06 ± 0.21 kcal min^−1^ during exercise. Energy balance was similar to HC (Fig. 1). Plasma glucose concentrations were unchanged, while plasma insulin was significantly higher, and plasma NEFA lower than in F (Fig. 2).

## Discussion

Ketogenesis is quantitatively small after an overnight fast, yet basal acetone concentration could be quantified in all breath samples. Furthermore, breath acetone showed significant changes over a 6-h period according to the feeding status and energy balance. When subjects remained fasted, the cumulated energy balance was slightly negative and breath acetone and plasma BOHB increased over time. In contrast, breath acetone and plasma BOHB slightly decreased when subjects were fed HC meals. This indicates that ketogenesis is activated when energy balance is negative, but is suppressed by positive energy balance associated with HC meals.

Our study also identifies limitations to the use of breath acetone as a marker of energy balance, however. We indeed observed that breath acetone concentrations increased when volunteers achieved a positive energy balance by ingestion of LC rather than HC meals. Lipid oxidation and plasma NEFA concentrations were also higher with LC than HC meals, consistent with a lesser suppression of ketogenesis. We also observed that a two-hour negative energy balance induced by exercise did not accelerate the rise in breath acetone concentrations in fasted subjects and did not increase it acutely when subjects were fed small HC meals. This suggests that stimulation of ketogenesis may be somewhat delayed relative to the beginning of a low intensity exercise.

Our results also point to some unexpected aspects of ketone bodies’ metabolism. Changes in breath acetone were correlated with changes in blood BOHB in F, but not in HC and LC. Furthermore, in LC, breath acetone increased, but blood BOHB decreased. This may be explained by the too often unrecognized complexity of ketone bodies’ metabolism. Ketogenesis initially yields acetoacetate, which is subsequently either reduced to BOHB or decarboxylated to acetone. BOHB and acetone are then oxidized through distinct pathways in extrahepatic tissues^11,12^. Acetone and BOHB concentrations will therefore vary according, not only to changes in acetoacetate production rate, but also to the relative changes of acetoacetate conversion into BOHB and acetone, and of BOHB and acetone oxidation. It is therefore possible that consumption of a very low-carbohydrate diet favored acetone over BOHB formation, or increased BOHB oxidation relative to that of acetone. This may be related to a the small postprandial insulin secretion after LC meals, since insulin was shown to increase BOHB clearance and oxidation^13^.

In conclusion, our study indicates that breath acetone is present in detectable amounts in normal subjects, and increases over time when subjects remain fasted, but not when they are fed carbohydrate containing meals. However, it is not suppressed by very low-carbohydrate meals, which may limit its use as a marker of energy balance in subjects on special diets.
